# Sucrose Promotes Strawberry Fruit Ripening and Affects Ripening-Related Processes

**DOI:** 10.1155/2019/9203057

**Published:** 2019-11-20

**Authors:** Ya Luo, Yuanxiu Lin, Fan Mo, Cong Ge, Leiyu Jiang, Yong Zhang, Qing Chen, Bo Sun, Yan Wang, Xiaorong Wang, Haoru Tang

**Affiliations:** ^1^College of Horticulture, Sichuan Agricultural University, Chengdu, Sichuan 611130, China; ^2^Institute of Pomology & Olericulture, Sichuan Agricultural University, Chengdu, Sichuan 611130, China

## Abstract

Strawberry is a typical nonclimacteric fruit, whose ripening mechanism needs to be further investigated. Sucrose has been recently proved as a signal molecule, participating in strawberry fruit ripening and related processes. While in the effects of sucrose application timing and concentration on ripening, fruit qualities remain unclear, as well as the transcriptome-wide details about the effects of sucrose on the gene expression involved in ripening-related processes. In this study, strawberry fruits at the degreening (DG), white (W), and initial-red (IR) stages were treated with different concentration of sucrose. The results showed that anthocyanin was increased while total polyphenol concentration (TPC) and total flavonoid concentration (TFC) were decreased during fruit development after sucrose treatment. Interestingly, It was showed that 100 mM sucrose application at the DG stage had the most obvious effects on fruit ripening; it made all the fruits turn into full-red (FR) around 4 days (d) earlier than the control, while it did not affect fruit quality traits and most bioactive compounds in the FR fruits. Subsequently, RNA sequencing (RNAseq) of the fruits collected at 8 days after 100 mM sucrose treatment was carried out. It was suggested that 993 genes were differentially expressed comparing with the control. Transcriptome-based expression analysis revealed that sucrose induced the expression of genes involved in the AsA and anthocyanin biosynthesis, while largely suppressed the expression of genes in TCA. The results obtained in this study provided more expression profiles of ripening-related genes under the treatment of sucrose, which will contribute to a better understanding for the mechanism underlying sucrose-induced fruit ripening.

## 1. Introduction

Fruit ripening is a complex developmentally regulated and genetically controlled process, which activates a whole set of biochemical and physiological pathways [1–4]. According to the ethylene production and their physiological differences in respiratory mode during ripening, fleshy fruits are generally classified as climacteric and nonclimacteric. In climacteric fruit such as tomato, kiwifruit, apple, and banana, numerous research data have been accumulated on the major regulation of ripening process by ethylene [5–7]. By contrast, in nonclimacteric fruits, ripening process does not depend on ethylene [8, 9], and the nature of the triggers of ripening in this type of fruit remains yet to be elucidated.

As a typical nonclimacteric flesh fruit, strawberry is not only an important healthy diet for consumers but also a model resource for researchers underlying the mechanism of fruit ripening. Over the past decades, it has been suggested that sucrose, which is generally known as an important metabolic resource and structural component supplier, is involved in regulating various plant processes including photosynthesis [10, 11], secondary metabolism, and hormonal balance [12]. Recently, sucrose has been suggested as a signal molecule able to control gene expression [13] and involved in the regulation of fruit ripening [14]. The expression of starch synthase and *β*-amylase genes was induced by sucrose [15, 16]. Exogenous sucrose accelerated the ripening process of postharvest tomato fruit and upregulated the expression of ethylene biosynthesis genes [17]. It could also induce anthocyanin accumulation, which is the most obvious trait of fruit ripening [18–20]. In strawberry, sucrose dramatically accelerated fruit coloring [21], while silencing of sucrose transporter gene *FaSUT1* had prevented the ripening process [14]. It was further found that sucrose could promote strawberry fruit ripening by modulating the expression of ripening-related genes [14], ABA biosynthetic gene *FaNCED1*, and through ABA-stress ripening transcription factor *ASR* [22]. However, the details about the effects of exogenous sucrose application timing and concentration on fruit coloring, fruit quality, bioactive compound content, and the expression of genes involved in ripening processes need to be further explored. In the present study, strawberry fruit at the DG, W, and IR stages was treated with 50 mM, 100 mM, and 150 mM sucrose, respectively; the coloring, quality, and bioactive compound content of the fruits were analyzed. Subsequently, the 100 mM sucrose-treated strawberry fruits were used for RNAseq; the effects of sucrose on the expression of genes involved in ripening-related processes including AsA, anthocyanin, sugars, and starch metabolism were assessed. The results would be a benefit to the underlying mechanism of strawberry fruit ripening, especially under the regulation of sucrose, with the purpose of improving the fruit quality.

## 2. Materials and Methods

### 2.1. Plant Materials and Treatment

Strawberry (*Fragaria*×*ananassa* cv. Benihoppe) fruits were planted in a greenhouse in Chengdu, China. In total, approximately 3,000 secondary flowers from at least 700 plants were tagged, and the fruits at the DG (18 days after anthesis, DPA), W (20 DPA), and IR (23 DPA) stages [21] were sprayed with 50 mM, 100 mM, and 150 mM sucrose solution, respectively, until dripping; fruits sprayed with water (0 mM sucrose) were used as control. The processing of fruit coloring was recorded at 4-day intervals from the beginning of treatment, and 15 berries per treatment at 8 days after the treatment were randomly sampled for fruit physiochemical analyses and RNAseq. All the berries for each treatment were sampled individually with three biological replications.

### 2.2. Fruit Physiochemical Analysis

Fruits were weighed using the electronic balance (Sartorius®), and fruit vertical and the horizontal diameters were measured by a vernier caliper. Fruit color was determined by a chroma meter (CR-400, Konica Minolta, Japan) with color characteristics—*L*^∗^, *h*°, and *C*^∗^ at three different parts around the equatorial region of each fruit. Total soluble solids (TSS) were determined using a refractometer (PAL-1, Atago Co. Ltd., Japan) while the titratable acid (TA) content was determined by repeated titrations with 0.1 mol·L^−1^ NaOH to a faint pink and the citric acid content was estimated as described previously.

The pH differential method was adopted for the total anthocyanin determination [23]. Briefly, the mixed strawberry fruit (1.0 g) was extracted with 5 mL of cold 1% HCl-ethanol and centrifuged at 9,000 × *g* for 15 min at 4°C, and then the supernatants were used for measuring the total anthocyanin content. The results were shown as milligram of pelargonidin 3-glucoside equivalents per 100 g of fresh weight.

The AsA measurements were based on the method of Sun et al. [24]. About 5.0 g of mixed strawberry fruit was extracted using 30 mL of 5% (*w*/*v*) metaphosphoric acid, followed by centrifugation at 22,000 × *g* for 15 min and quantified at 525 nm for AsA. Results were expressed as mg of AsA per 100 g of fresh weight.

To determine TPC and TFC, approximately 5.0 g of mixed strawberry fruit was extracted with 25 mL of 80% acetone for 1 h at room temperature, followed by centrifugation (10 min, 4,500 × *g*) at room temperature, and the supernatant was collected for the measurement according to Molan et al. [25] and Chang et al. [26], respectively. TPC and TFC were exhibited as g gallic acid and quercetin equivalents per 100 g of fresh weight, respectively. The above experiments were repeated three times.

### 2.3. RNA Extraction and Sequencing

Total RNA was isolated from the fruits at 8 days posttreatment using the improved CTAB method [27] and subsequently incubated with RNase-free DNase I for 30 min to remove the genome DNA pollution. Concentration and integrity of each RNA sample were assessed by a NanoDrop 1000 spectrophotometer and an Agilent 2100 bioanalyzer. The samples with concentration above 400 ng/*μ*L, RIN (RNA integrity number) values above 8, and the OD of 260/280 and 260/230 ratios above 1.8 were selected for library construction. Libraries were constructed and sequenced by the Beijing Genomics Institute (BGI, Shenzhen, China) using an Illumina HiSeq™ 2000 platform. Three independent libraries as three biological replicates were sequenced for each sample.

### 2.4. Transcript Assembly and Expression Quantification

Considering the incompletion of the cultivated strawberry (*Fragaria×ananassa*) genome and the potential difference between diploid and octoploid *Fragaria* species, the clean reads were *de novo* assembled using Trinity software (version 2.4.0) [28] with the parameters of “min_kmer_cov = 2, normalize_reads.” The assembled nonredundant transcripts were further annotated by aligning against to protein databases including swiss-prot (http://www.uniprot.org) and UniRef90 (https://www.uniprot.org/help/uniref) using Diamond BLASTx [29]. Transcript expression levels were quantified and normalized by the fragments per kilobase million (FPKM) values using RSEM [30]. DESeq2 R package [31] was applied for differential expression analysis. Significantly differentially expressed genes (DEGs) were defined with an absolute value of log2 fold change (FC) ≥ 1 and FDR < 0.05.

### 2.5. GO and KEGG Enrichment Analysis

GO annotation was performed by aligning on UniRef90 database using a specific perl script. Fisher test enrichment calculation was performed by the TopGo R package [32] using the whole assembled transcripts as background. Kyoto Encyclopedia of Genes and Genomes (KEGG) enrichment analysis was carried out by Kobas 3.0 software [33]. The results of enrichment analysis of DEGs were visualized by the R ggplot function.

### 2.6. Statistical Analysis

The data were analyzed by the one-way ANOVA test using SPSS software (version 17.0; IBM, USA) and were expressed as mean ± standard error. A *P* value of ≤0.05 was considered statistically significant difference.

## 3. Results

### 3.1. Effects of Exogenous Sucrose on Strawberry Fruit Coloring

**Figure 1:**
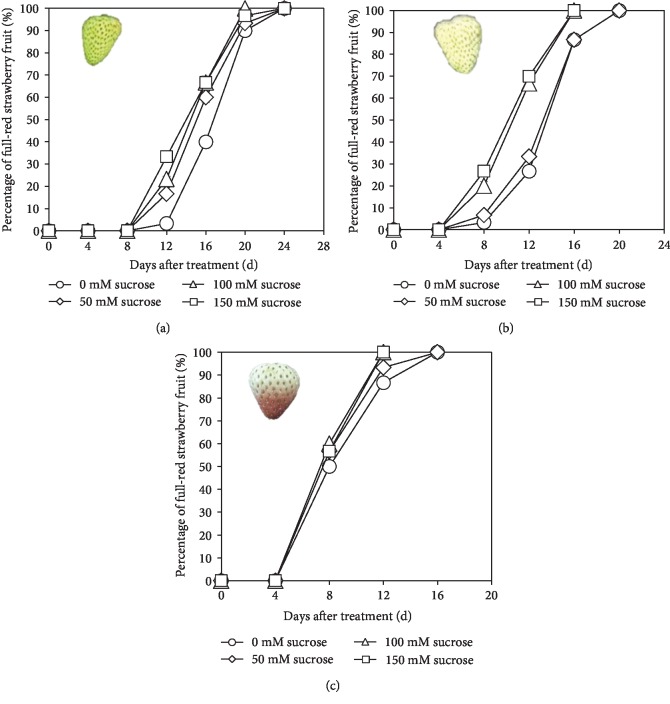
Percentage of FR strawberry after application of different concentrations of sucrose at the (a) DG, (b) W, and (c) IR stages. Each data point represented the mean value of three replicate recordings.

Exogenous sucrose could accelerate fruit coloring depending on the application timing and concentration of sucrose (Figure 1). When the fruits were treated at the DG stage, 50, 100, and 150 mM sucrose could make the fruits turn into FR faster than the control. Particularly, the percentage of FR fruits on the 12 d and 16 d after treatment increased by 400.06%, 600.6%, and 900.9%, respectively, compared with the control. Interestingly, the 100 mM sucrose application made all the fruits turn into FR on the 20^th^ d after treatment, which was 4 d earlier than the other two treatments and control, as they made all the fruits reach the FR stage on the 24^th^ d after application (Figure 1(a)). When the fruits were treated at the W stage, higher sucrose concentration had more obvious effects on accelerating fruit coloring. As shown in Figure 1(b), 100 mM and 150 mM sucrose could speed the fruit coloring by making all the fruits reach the FR stage on the 16^th^ d after treatment. By contrast, 50 mM sucrose treatment had no significant effects on fruit coloring; it made all the fruits turn into FR on the 20^th^ d after the treatment, which was the same with control (Figure 1(b)). Similarly, when fruits were treated at the IR stage, 100 mM and 150 mM sucrose application could make all the fruits reach FR around 4 d earlier than the control (Figure 1(c)).

### 3.2. Effects of Exogenous Sucrose on FR Fruit Weight and Appearance Quality

Although sucrose treatment advanced the fruit harvest date, the fruit weight was not affected except for the fruits treated with 150 mM sucrose at the DG stage (Table 1). Exogenous application of 10 mM, 50 mM, and 100 mM sucrose also had almost no effect on fruit horizontal diameters and fruit shape index, while it increased the vertical diameter fruits treated at the DG stage by 12.43%, 7.30%, or 11.89%, respectively, compared with control (Table 1). The lightness, color shade, and saturation of the strawberry fruit are three important factors determining the commercial value of strawberry. They were measured and reflected by *L*^∗^ values, *h*° values, and *C*^∗^ values. The *h*° values and *C*^∗^ values between the control and treated fruits showed no significant differences while the *L*^∗^ values exhibited a reduction, particularly in fruits treated with 150 mM sucrose at the W or IR stage (Table 1).

### 3.3. Effects of Exogenous Sucrose on FR Fruit Nutritional Quality and Bioactive Compound Contents

**Figure 2:**
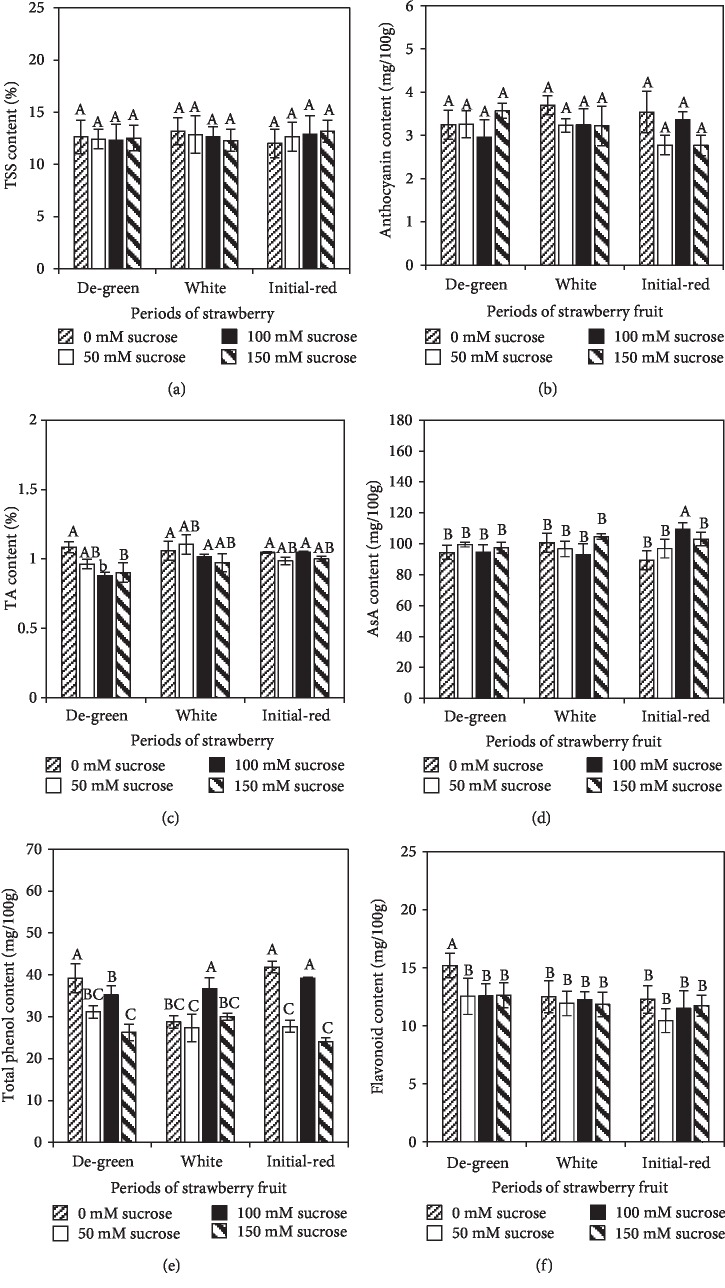
Strawberry fruit nutritional quality and bioactive compound content after 50 mM, 100 mM, or 150 mM sucrose application at DG, W, and IR stages. 0 mM sucrose indicated control fruits treated with water. Bars represented the mean values ± stand error. (a) Total soluble solids, (b) anthocyanin content, (c) titratable acidity content, (d) AsA content, (e) total phenol content, and (f) flavonoid content in full-red strawberry fruit under different treatments. Different letters on the bars represent the significance at the *P* ≤ 0.05 level.

In terms of the fruit nutritional quality, exogenous sucrose treatment did not affect the TSS (Figure 2(a)) and anthocyanin content (Figure 2(b)) but decreased the TA content (Figure 2(c)) particularly in fruit treated at the DG stage. In addition, among all the treatments, only the application of 100 mM sucrose at the IR stage increased the AsA content by 8.47% compared with the control (Figure 2(d)). Furthermore, in comparison with the control fruit, in the fruit treated at the DG and IR stages, the TPC was decreased by 20.57%, 33.04% and 33.91%, 42.55%, respectively, under 50 mM and 150 mM sucrose application. By contrast, it showed no obvious difference between the control and fruits treated with 100 mM sucrose at the DG or IR stage (Figure 2(e)), while it was increased by 27.66% in the fruit when treated at the W stage (Figure 2(e)). TFC was decreased by 17.37%, 17.11%, and 16.78% in the fruit treated with 50 mM, 100 mM, and 150 mM sucrose at the DG stage, while it showed no significant difference between the control and fruits treated at the W or IR stage (Figure 2(f)).

### 3.4. Effects of Exogenous Sucrose on AsA, Anthocyanin, and Bioactive Compounds during Fruit Development

According to the above results, together with the consideration of the positive effects on accelerating fruit coloring, maintaining fruit quality and the bioactive compound content, fruits at the DG stage were treated with 100 mM sucrose and fruits at 8 days after the treatment were collected for further analysis.

**Figure 3:**
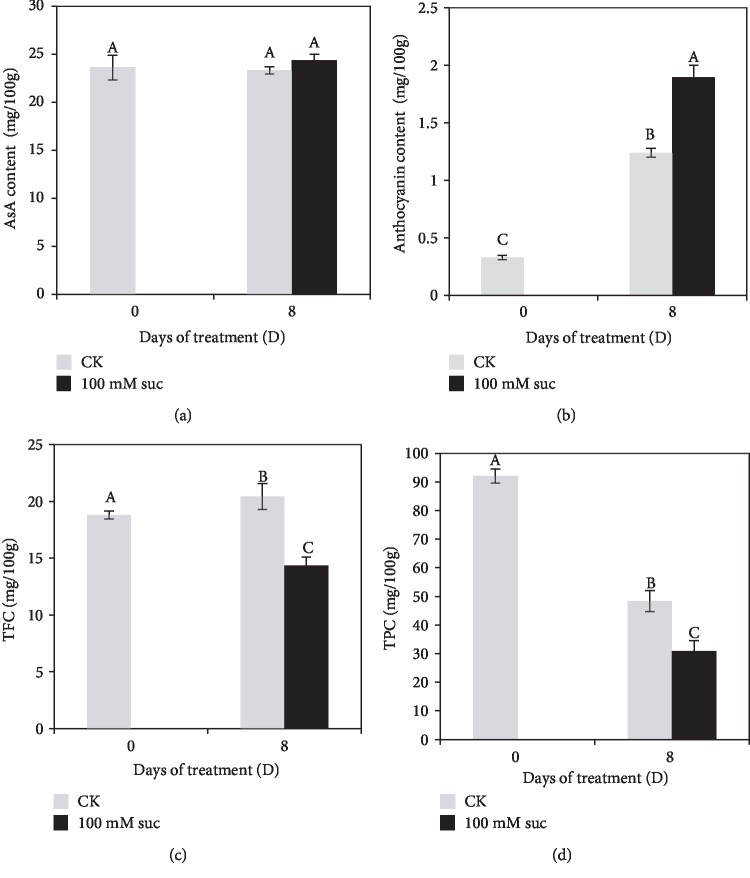
The content of ascorbic acid (AsA) (a), anthocyanin (b), total flavonoid concentration (TFC) (c), and total polyphenol concentration (TPC) (d) in strawberry fruit on the 8^th^ day after 100 mM sucrose application. Different letters on the bars represent the significance at the *P* ≤ 0.05 level.

The results showed that the content of anthocyanin and TFC increased, while AsA showed no difference and TPC decreased gradually with the development (8 days after the DG stage) of strawberry fruit (Figure 3). Exogenous sucrose treatment accelerated the accumulation of AsA (Figure 3(a)) and anthocyanin (Figure 3(b)) but decreased the TFC (Figure 3(c)). The anthocyanin content and TFC were increased by 53.23% and decreased by 29.76%, respectively, compared with the control. Furthermore, sucrose treatment prompted a rapid reduction of TPC in fruit at the 8 days after application, with decreases of 36.08%, when compared with the control (Figure 3(d)).

### 3.5. RNAseq, Differential Expression Analysis, and KEGG Enrichment

**Figure 4:**
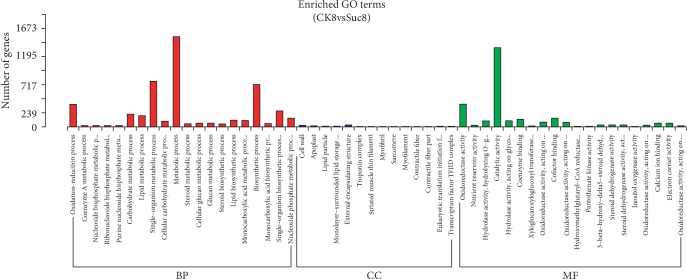
Classification of enriched GO terms of DEGs comparing the sucrose-treated fruits with control fruits. CK8: control fruits on the 8^th^ day after treated with water; Suc8: fruits on the 8^th^ day after treated with sucrose; BP: biological process; CC: cellular component; MF: molecular function.

To generate more details about the effects of sucrose on the gene expression, RNAseq was carried out using the fruits at 8 days after 100 mM sucrose treatment. Based on our transcriptome data, 993 genes were differentially expressed in the sucrose-treated fruits comparing with the control fruits. Among them, 558 genes were upregulated and 435 were downregulated. GO enrichment analysis of GO categories according to biological process (BP), molecular function (MF), and cellular component (CC) was performed to further elucidate the functions of DEGs. As shown in results (Figure 4), among the BP terms, metabolic process, single-organism metabolic process, and biosynthetic process were the most enriched. Notably, oxidation-reduction process was enriched as well. Correspondingly, a large number of DEGs were enriched into catalytic activity and oxidoreductase activity MF terms, suggesting that the function of DEGs might be related to oxidative activity. In addition, the most frequent CC terms indicated that the DEGs in the sucrose-treated fruits preferentially function in the extracellular region such as cell wall and external encapsulating structure.

**Figure 5:**
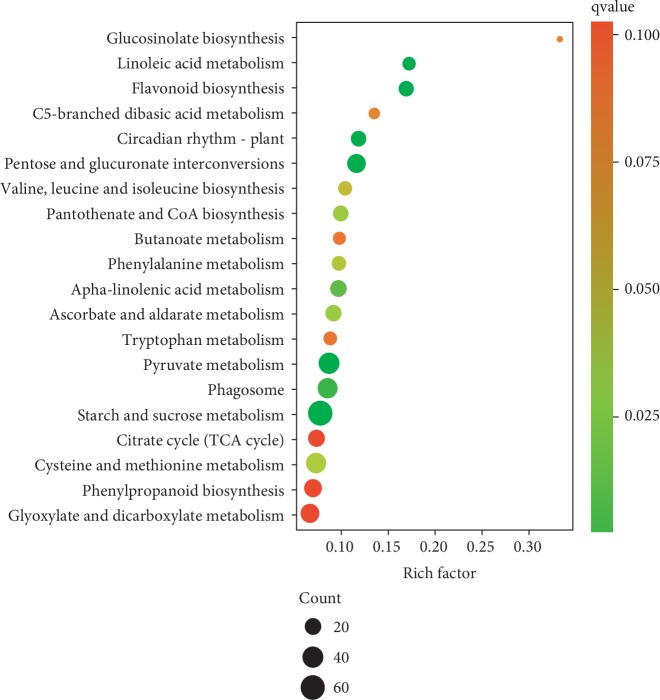
Top 20 enriched KEGG pathways of DEGs in sucrose-treated and control fruits. The *Y*-axis on the left represents KEGG pathways; the *X*-axis indicates the enrichment factor. Rich factor means the ratio of differential expressed genes and all annotated genes in one pathway. Low qvalues are shown in green, and high qvalues are depicted in red. Pathways with qvalue less than 0.05 are significantly enriched. The size of the spot represents the number of DEGs, and the color of the spot corresponds to different qvalue ranges.

Furthermore, to investigate the potential effects of sucrose on pathways, KEGG enrichment analysis of DEGs was carried out. Our results (Figure 5) revealed that DEGs were significantly enriched in the ripening-related metabolic process such as starch and sucrose metabolism, phenylalanine metabolism, and ascorbate and aldarate metabolism. These results gave us a clue for the effects of sucrose on the gene expression of ripening-related processes.

### 3.6. Expression Profiles of Genes Related to AsA and Glutathione Metabolism

**Figure 6:**
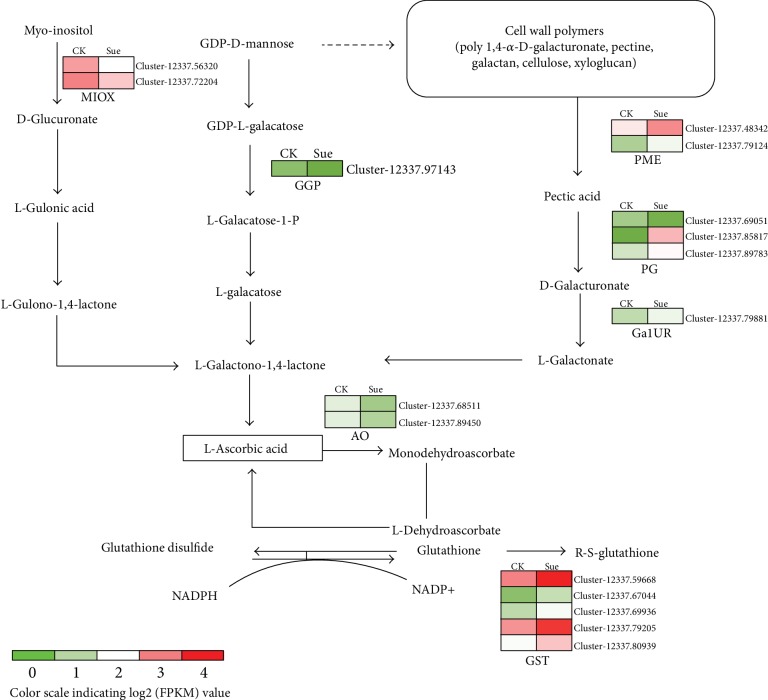
Schematic of AsA and glutathione metabolism pathway in strawberry. Expression patterns of DEGs were represented by a log2 FPKM value and showed as a heat map at the side of each step. CK represents the control fruits treated with water, Suc represents experimental fruits at the 8^th^ day after sucrose treatment. MIOX: inositol oxygenase; GGP: GDP-L-galactose phosphorylase; PME: pectinesterase; PG: polygalacturonase; GalUR: aldo-keto reductase; AO: L-ascorbate oxidase; GST: glutathione S-transferase.

To deeply explore the molecular effects of sucrose on the AsA metabolism, the expression profiling of transcripts involved in AsA biosynthesis was analyzed based on the transcriptome data. The results showed that several transcripts encoding enzymes involved in the alternative D-glucuronate pathway for the AsA biosynthesis pathway were upregulated (Figure 6, Table 1). For instance, transcript Cluster-12337.79881 encoding aldo-keto reductase expressed 2 folds higher in sucrose-treated fruits than that in the control fruits. The expression levels of 2 transcripts encoding pectinesterase (PME) and 3 transcripts encoding polygalacturonase (PG) involved in the early steps of the D-galacturonate biosynthesis pathway were also enhanced at least 2-fold in sucrose-treated fruits. Our results suggested a higher flux of the D-galacturonate biosynthesis pathway for AsA accumulation. However, notably, the expression of one transcript (Cluster-12337.97143) encoding GDP-L-galactose phosphorylase (GGP, VTC2/VTC5) involved in the predominant AsA biosynthetic pathway (Wheeler-Smirnoff pathway) was largely inhibited in the sucrose-treated fruits with log2 fold change value about -2.6, whereas transcripts encoding other key enzymes involved in this predominant pathway showed no differences in expression, such as GDP-D-mannose pyrophosphorylase (GMP). In addition, the transcript level of inositol oxygenase (MIOX) was also repressed in the sucrose-treated fruits. What is more, several transcripts encoding enzymes in the glutathione metabolism were upregulated under sucrose treatment, including glutathione-S-transferase (GST) and ribonucleoside-diphosphate reductase (RDR), reflecting a higher flux in glutathione metabolism in sucrose-treated strawberry.

### 3.7. Expression of Transcripts Related to Anthocyanin Metabolism

**Figure 7:**
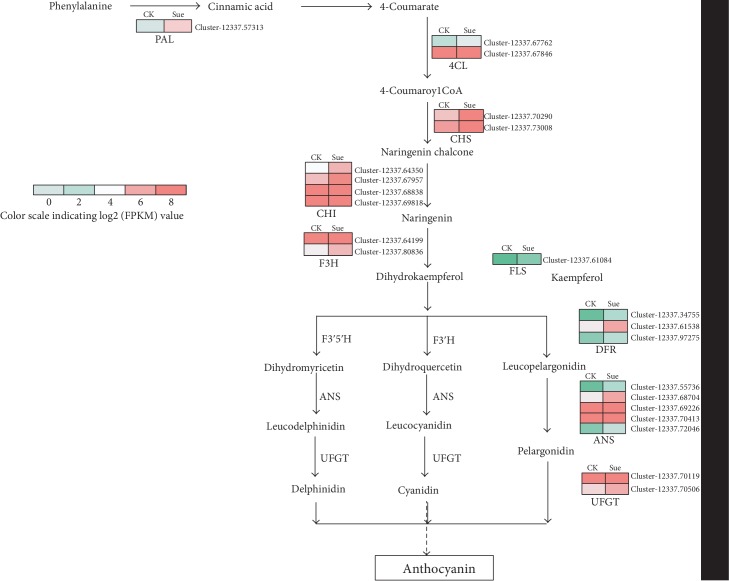
Effects of sucrose on the expression of genes encoding flavonoid and anthocyanin biosynthetic enzymes in strawberry. Expression patterns of DEGs were represented by a log2 FPKM value and showed as a heat map at the side of each step. CK represents the control fruits treated with water, Suc represents experimental fruits at the 8^th^ day after sucrose treatment. PAL: phenylalanine ammonia-lyase; 4CL: 4-coumarate-CoA ligase; CHS: chalcone synthase; CHI: chalcone-flavonone isomerase; F3H: flavanone 3-hydroxylase; FLS: flavonol synthase; DFR: dihydroflavonol-4-reductase; ANS: anthocyanidin synthase; UFGT: flavonoid 3-O-glucosyltransferase.

The phenylalanine and flavonoid metabolisms were significantly enriched in the DEGs, indicating that sucrose could affect the anthocyanin metabolism. Thus, the expression profiles of transcripts involved in flavonoid and anthocyanin metabolisms were analyzed in detail to investigate the effects of sucrose on anthocyanins. The results (Figure 7) showed that the expression levels of transcripts involved in the anthocyanins (including the early and late steps) were largely increased under the sucrose treatment. For instance, the transcript Cluster-12337.57313 encoding phenylalanine ammonia-lyase (PAL) and Cluster-12337.67762 encoding 4-coumarate-CoA (4CL) expressed 4.6 and 2.3 folds higher, respectively, in the sucrose-treated fruits comparing with the control (Table 1). In the late steps, the expressions of transcripts encoding anthocyanidin synthase (ANS) and flavonoid 3-O-glucosyltransferase (UFGT) were both increased at least 2 folds ([Supplementary-material supplementary-material-1]). These results indicated that sucrose could enhance the accumulation of anthocyanins.

### 3.8. Expression of Transcripts Related to Starch, Sucrose, Glycolysis, and TCA Metabolism

**Figure 8:**
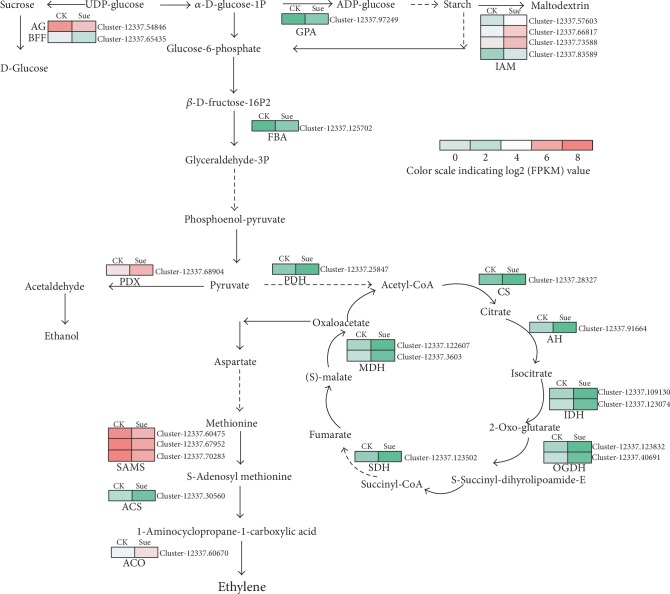
Effects of sucrose on the expression of genes encoding enzymes involved in sucrose, starch, glycolysis, TCA cycle, and ethylene biosynthesis pathways in strawberry. Expression patterns of DEGs were represented by a log2 FPKM value and showed as a heat map at the side of each step. CK represents the control fruits treated with water, Suc represents experimental fruits at the 8^th^ day after sucrose treatment. AG: alpha-glucosidase; BFF: acid beta-fructofuranosidase; GPA: glucose-1-phosphate adenylyltransferase; IAM: isoamylase; FBA: fructose-bisphosphate aldolase; PDX: pyruvate decarboxylase; PDH: pyruvate dehydrogenase; CS: citrate synthase; AH: aconitate hydratase; IDH: isocitrate dehydrogenase; OGDH: 2-oxoglutarate dehydrogenase; SDH: succinate dehydrogenase; MDH: malate dehydrogenase; SAMS: S-adenosylmethionine synthase; ACS: 1-aminocyclopropane-1-carboxylate synthase; ACO: 1-aminocyclopropane-1-carboxylate oxidase.

To further elucidate the effects of sucrose on ripening-related carbohydrate catabolism, the expression levels of transcripts involved in sucrose, starch, glycolysis, and citrate acid (TCA) cycle were established. The results (Figure 8) showed that glucosidase (AG) and fructofuranosidase (BFF) catalyzing the transformation of sucrose to glucose expressed lower, while the enzymes involved in the starch branch including glucose-1-phophate adenylyltransferase (GPA) and isoamylase (IAM) were increased in sucrose-treated fruits comparing with the control. Notably, the expression of the key enzyme fructose-bisphosphate aldolase in the glycolysis pathway was largely enhanced with the log2 fold change (sucrose-treated/control) value about 4.2. In contrast, several enzymes involved in TCA such as citrate synthase, isocitrate dehydrogenase, and malate dehydrogenase showed decreasing in expression. In addition, the expression levels of key enzymes involved in ethylene synthesis including S-adenosylmethionine synthase (SAMS) and 1-aminocyclopropane-1-carboxylate synthase (ACS) were also significantly impressed by sucrose. These results indicated that sucrose might affect ripening-related process through altering the expression of corresponding transcripts.

## 4. Discussion

### 4.1. Exogenous Sucrose Accelerating Fruit Ripening and Anthocyanin Biosynthesis

Fruit coloring is the one of the most obvious traits of fruit ripening, which is because of the anthocyanin accumulation in strawberry. Anthocyanin is regulated by its biosynthetic genes, transcription factors, and noncoding RNAs [34]. Sucrose-induced anthocyanin accumulation has also been observed in many plant species. Jia et al. [14] have previously suggested that sucrose treatment could accelerate strawberry fruit coloring; it could strongly enhance the flavonoid and anthocyanin contents in *Arabidopsis* as well [35]. On the transcript level, it has been suggested that the expression of grape *DFR* was proved to be responsive to sucrose [36], and the expression of genes involved in the whole biosynthesis pathways was strongly upregulated by sucrose treatment [37]. Identically, our results showed that sucrose treatment could advance the fruit ripening, as they turned to FR 4 days earlier than the fruit treated with water. In addition, the anthocyanin content was significantly enhanced during fruit ripening in sucrose-treated fruits comparing with the control. This is because the expression levels of transcripts encoding the anthocyanin biosynthetic enzymes were largely upregulated as our results showed (Figure 7). However, the anthocyanin content in the FR fruits was not significantly changed by sucrose application, indicating that sucrose could speed the ripening process without changing the final anthocyanin concentration.

### 4.2. Sucrose Suppressed the TCA Cycle

Ripening-related processes require both energy and a supply of carbon skeleton building blocks, which are supplied by respiration metabolism [38]. The glycolysis and tricarboxylic acid (TCA) cycle are commonly thought as two major respiratory metabolism pathways, playing an essential role in providing energy (ATP), reducing power (NAD(P)H) and precursors for series secondary metabolite biosynthesis [39, 40]. It has been previously suggested that overexpression of sucrose phosphorylase resulted a reduction of sucrose accompanying with an increase of glycolysis [41]. However, in our results, sucrose treatment did not affect the glycolysis pathway in strawberry except for the expression of transcripts encoding fructose-bisphosphate aldolase, which was largely upregulated. It is known that glycolysis and TCA cycle are linked by the pyruvate dehydrogenase, which is responsible for the conversion of pyruvate to acetyl-CoA. Intriguingly, we found that the transcript (Cluster-12337.25847) encoding E1 component subunit alpha of pyruvate dehydrogenase gene (*PDHE1á*) was significantly downregulated (log2 fold change (sucrose/ck) -3.75) in the sucrose-treated fruits. This might contribute to the sucrose-induced ripening, which is supported by the results that the downregulation of *PDHE1á* accelerated strawberry fruit ripening [42]. They further found that this enhancement was due to the inhibition of respiration and ATP synthesis. In addition, our results also showed that most of the enzymes in the downstream TCA cycle exhibited downregulation in transcript levels, suggesting an inhibition of TCA cycle. On the one hand, this could lead to the decreasing of substrates of respiration and finally lead to respiratory inhibition, which might be helpful to explain the induction of ripening by sucrose. Because as generally known, sugars and organic acid are two major substrates of respiration [38]. While during fruit ripening, sugars are stored in the vacuole causing a shift from sugars to organic acid as respiratory substrate [43, 44]. On the other hand, this could contribute to the sucrose-induced ripening in the terms of fruit softening, since it has been proven that an inhibition of TCA cycle resulted in a restriction of cell wall biosynthesis [45].

### 4.3. Exogenous Sucrose Affects the Expression of Genes Involved in Ripening Process

Sucrose has been suggested as a key signal in the regulation of strawberry ripening, occurring via both ABA-dependent and ABA-independent pathways [14]. In ABA-independent way, sucrose signal could be received by sucrose transporter gene (SUT2) and then coupled with G protein to regulate the expression of genes involved in fruit ripening. Also, sucrose could be decomposed into fructose and glucose and then regulate the gene expression or participate in cell wall metabolism through affecting pectin [46, 47]. In our results, no changes in the expression of SUT2 were observed, while the genes encoding enzymes catalyzing the transformation of sucrose to fructose and glucose decreased. On the contrary, the genes involved in pectin metabolism including PME and PG increased ([Supplementary-material supplementary-material-1]). In ABA-dependent way, it was previously suggested that exogenous sucrose could induce the expression of 9-cis-epoxycarotenoid dioxygenase (NCED) and beta-glucosidase (BG), which are two key enzymes for upregulation of ABA levels [14, 21]. In our results, sucrose treatment had no effects on the expression of *FaNCED* but significantly upregulated the expression of *FaBG* ([Supplementary-material supplementary-material-1]), indicating that sucrose might enhance the ABA content. ABA could promote the interaction of *PYR* and *PP2C*, resulting in *PP2C* inhibition and *SnRK2* activation [48]. This is supported by our results that the expression levels of *PYR*/*PYL* and *PP2C* increased and decreased, respectively, in the sucrose-treated fruits. The difference is that the expression of *SnRK2* showed no change, which is consistent with another previous reports that sucrose treatment had no effect on the level of *SnRK2* expression [49], suggesting the complex regulation of *SnRK2* expression.

Additionally, sucrose could also interact with other hormones in regulating fruit ripening. For instance, it has been proven that exogenous sucrose could upregulate the expressions of ethylene biosynthetic genes and promote ethylene signal transduction in tomato fruit [17]. On the contrary, our results showed that sucrose treatment downregulated the expressions of ethylene-related genes including the biosynthesis genes like *SAM*S and *ACS* and the signaling genes including mitogen-activated protein kinase kinase (*SMKK*) and ethylene-responsive transcription (*ERF*) ([Supplementary-material supplementary-material-1]). This might be because of the different ripening patterns between climacteric and nonclimacteric fruits, as ripening of the nonclimacteric fruit does not depend on ethylene. However, a previous study suggested that sucrose had no more effect on the IAA gene expression [50]; our results showed that sucrose treatment downregulated the auxin and jasmonic acid signal transduction while upregulated the salicylic acid signaling.

In terms of the ripening-related AsA metabolism, based on our transcriptome data, the expression levels of *GGP* involved in the L-galactose biosynthesis pathway were largely decreased under sucrose treatment (Figure 6), which might lead to a decrease in AsA accumulation. Although the effects of sucrose on the expression of *GGP* has not been described before, the sucrose treatment could alter the expression of genes related to AsA metabolism [51]. On the contrary, the expression levels of transcripts encoding enzymes involved in the predominant D-galacturonate biosynthesis pathway were significantly upregulated by sucrose treatment, including *PME*, *PG*, and *GalUR*, resulted in a little increase in AsA content (Figure 3) although it was not statistic significantly. This might be because our measurements were only 8 d from the treatment, and there are probably not enough time elapsed to increase a lot. Taken together, our results showed that sucrose could affect the plant hormone signal transduction and ripening-related gene expression, which supplied the details of the effects of sucrose on ripening, while the interaction between sucrose and plant hormone need further exploration.

## Figures and Tables

**Table 1:** Effects of exogenous sucrose on FR fruit weight and appearance quality.

Treated stage	Sucrose concentrations (mM)	Weight (g)	Horizontal diameters (cm)	Vertical diameters (cm)	Fruit shape index	*L* ^∗^	*C* ^∗^	*h*°
DG	0	14.42 ± 4.46^b^	3.08 ± 0.39^a^	3.70 ± 0.38^b^	1.21 ± 0.01^a^	38.94 ± 7.22^b^	50.18 ± 5.89^ab^	32.08 ± 7.67^ab^
	50	16.79 ± 4.07^ab^	3.25 ± 0.26^a^	4.16 ± 0.29^a^	1.24 ± 0.03^a^	42.00 ± 8.91^a^	50.61 ± 5.91^ab^	34.78 ± 8.82^a^
	100	17.04 ± 3.75^ab^	3.25 ± 0.23^a^	3.97 ± 0.34^a^	1.26 ± 0.05^a^	40.31 ± 7.20^ab^	52.31 ± 5.02^a^	34.03 ± 8.48^ab^
	150	18.23 ± 5.18^a^	3.30 ± 0.38^a^	4.14 ± 0.47^a^	1.26 ± 0.04^a^	40.82 ± 8.27^ab^	49.71 ± 5.89^ab^	34.25 ± 9.25^ab^

W	0	19.37 ± 5.25^a^	3.36 ± 0.34^a^	4.09 ± 0.40^a^	1.22 ± 0.03^a^	42.66 ± 9.12^a^	49.57 ± 4.62^b^	34.60 ± 8.83^a^
	50	19.18 ± 3.48^a^	3.31 ± 0.37^a^	4.09 ± 0.31^a^	1.23 ± 0.02^a^	41.78 ± 7.98^ab^	51.74 ± 7.44^ab^	33.94 ± 9.72^ab^
	100	18.23 ± 5.57^a^	3.32 ± 0.37^a^	4.09 ± 0.55^a^	1.23 ± 0.02^a^	41.46 ± 7.94^ab^	50.86 ± 4.75^ab^	33.74 ± 7.82^ab^
	150	19.03 ± 3.48^a^	3.32 ± 0.38^a^	4.08 ± 0.29^a^	1.24 ± 0.03^a^	40.02 ± 6.82^b^	51.29 ± 4.26^ab^	32.43 ± 7.26^ab^

IR	0	19.78 ± 4.28^a^	3.32 ± 0.39^a^	4.13 ± 0.36^ab^	1.22 ± 0.04^a^	39.17 ± 5.18^b^	51.81 ± 3.59^a^	31.91 ± 6.10^ab^
	50	21.83 ± 6.96^a^	3.32 ± 0.40^a^	4.30 ± 0.50^a^	1.24 ± 0.02^a^	37.24 ± 4.89^b^	51.23 ± 4.33^a^	30.98 ± 5.20^ab^
	100	19.68 ± 5.61^a^	3.32 ± 0.41^a^	4.15 ± 0.48^ab^	1.24 ± 0.03^a^	38.16 ± 6.49^b^	50.90 ± 4.99^a^	32.02 ± 6.62^ab^
	150	19.57 ± 5.60^a^	3.32 ± 0.42^a^	4.16 ± 0.47^ab^	1.27 ± 0.06^a^	36.15 ± 5.56^c^	50.39 ± 5.75^a^	30.40 ± 5.38^b^

Note: values represented the means ± standard error. Lowercase letters represented the significance at the *P* ≤ 0.05 level in columns.

## Data Availability

The read count data used to support the findings of this study are included within the supplementary information file.

## Supplementary Materials

Supplementary Materials Table S1: list of genes involved in ripening-related processes affected by sucrose.
